# Malawian children with chest-indrawing pneumonia with and without comorbidities or danger signs

**DOI:** 10.7189/jogh.11.04016

**Published:** 2021-03-07

**Authors:** Amy Sarah Ginsburg, Tisungane Mvalo, Melda Phiri, Daphne Gadama, Claightone Chirombo, Madalitso Maliwichi, Jun Hwang, Susanne May

**Affiliations:** 1 University of Washington, Seattle, Washington, USA; 2 University of North Carolina Project, Lilongwe Medical Relief Fund Trust, Lilongwe, Malawi

## Abstract

**Background:**

Children with comorbidities or danger signs are often excluded from trials evaluating pneumonia treatment.

**Methods:**

We sought to investigate whether the percentage of children with chest-indrawing pneumonia cured at Day 14 was lower among those with HIV infection or exposure, malaria, moderate or severe acute malnutrition, or anemia enrolled in a prospective observational cohort study than among children without these comorbidities enrolled in a concurrent prospective randomized controlled trial evaluating duration of amoxicillin treatment in Lilongwe, Malawi.

**Results:**

Children with chest-indrawing pneumonia and comorbidities but without danger signs did not have statistically significant higher treatment failure rates by Day 6 than those in the chest-indrawing pneumonia clinical trial. However, children with chest-indrawing pneumonia and HIV infection or exposure, malaria, or moderate or severe acute malnutrition had higher rates of not being clinically cured at Day 14 when compared to children without these comorbidities (adjusted differences ranging from 7.7% to 17.0%). Furthermore, among children without danger signs at enrollment, but with HIV infection or HIV exposure or moderate or severe acute malnutrition, 12.5% and 15.6% respectively were not clinically cured at Day 14 even though they were without treatment failure by Day 6.

**Conclusions:**

More intensive follow-up of children with chest-indrawing pneumonia and comorbidities who do not have danger signs may be beneficial.

Globally, pneumonia remains the leading infectious cause of childhood mortality [1,2]. As part of the Innovative Treatments in Pneumonia (ITIP) project in Lilongwe, Malawi, a randomized controlled trial (ITIP2) was conducted to evaluate the optimal duration of treatment with amoxicillin for chest-indrawing pneumonia (CIP) [3,4]. In parallel, we also conducted an observational study (ITIP3) of CIP children who were excluded from ITIP2 due to selected comorbidities or World Health Organization (WHO) general or respiratory danger signs [5]. Pneumonia in children with comorbidities is common, and risk factors for poor outcomes can be linked to the child (eg, sex, age, underlying diseases), disease (eg, type of infection), environment, family and its socio-economic status, and health system and type of care [6]. In low- and middle-income countries, these risk factors include young age, malnutrition, anemia, HIV infection or exposure, chronic underlying diseases, air pollution, poor sanitation, overcrowding, low socio-economic status, young maternal age, low maternal education and limited access to health care [6,7]. These risk factors can influence whether exposure to an etiologic agent will produce a severe or even, deadly case of pneumonia [6-11]. To better understand pneumonia treatment outcomes among higher risk African children post-introduction of *Haemophilus influenzae* type b and *Streptococcus pneumoniae* conjugate vaccines, and to interpret the results of the concurrent clinical trial, we compare clinical outcomes of ITIP3′s CIP participants (ie, those with selected comorbidities and/or danger signs) to those in ITIP2 (ie, those without selected comorbidities or danger signs).

## METHODS

### Study design

An objective of the ITIP3 prospective observational study was to investigate whether the percentage of CIP children cured at Day 14 was lower among those with HIV infection or exposure, malaria, moderate acute malnutrition (MAM) or severe acute malnutrition (SAM), or anemia in ITIP3 than among the children in the standard-of-care arm in the concurrent ITIP2 prospective randomized controlled trial (ie, those without these selected comorbidities or without danger signs who were treated similarly) [4,5]. Children aged 2-59 months meeting the WHO case definition of CIP in the pediatric outpatient departments of Kamuzu Central Hospital (KCH) and Bwaila District Hospital (BDH) in Lilongwe, Malawi were screened by study staff to determine eligibility (**Table 1**). Those who were excluded from ITIP2 because of comorbidities (HIV infection or exposure, anemia with hemoglobin <8 g/dL), severe comorbidities (severe malaria, SAM), or WHO general or respiratory danger signs were assessed for enrollment into ITIP3.

**Table 1:** Study definitions, eligibility criteria, and clinical outcomes

Study definitions	
Fast-breathing pneumonia	Cough less than 14 days or difficulty breathing AND fast breathing for age
Chest-indrawing pneumonia	Cough less than 14 days or difficulty breathing AND visible indrawing of the chest wall with or without fast breathing for age
Fast breathing for age	Respiratory rate ≥50 breaths per minute (for children 2 to <12 months of age) or ≥40 breaths per minute (for children ≥12 months of age)
Very fast breathing for age	≥70 breaths per minute (for children 2 to <12 months of age) or ≥60 breaths per minute (for children ≥12 months of age)
Hypoxemia	Arterial oxyhemoglobin saturation (SpO_2_)<90% in room air, as assessed non-invasively by a pulse oximeter
Danger sign	Having 1 or more of the following:
	• World Health Organization (WHO) Integrated Management of Childhood Illness (IMCI) general danger sign, including lethargy or unconsciousness, convulsions, vomiting everything, inability to drink or breastfeed, or
	Severe respiratory distress, including grunting, nasal flaring, and/or head nodding
Major comorbidity	Having 1 or more of the following:
	• HIV infection or exposure
	• Malaria
	• Moderate or severe acute malnutrition
	• Anemia
HIV exposure	Children <24 months of age with a HIV-infected mother
Severe malaria	Positive malaria rapid diagnostic test (mRDT) result with any danger sign, stiff neck, abnormal bleeding, clinical jaundice, or hemoglobinuria
Laboratory-result malaria	Positive malaria RDT result without qualifying as severe malaria
Moderate acute malnutrition	Weight for height/length <-2 SD but ≥-3 SD, or mid-upper arm circumference (MUAC) <12.5 cm but ≥11.5 cm
Severe acute malnutrition	Weight for height/length <-3 SD, MUAC <11.5 cm, or peripheral edema
Anemia	Hemoglobin level <8 g/dL
Vaccination status	Either having received:
	• Age-appropriate number of doses with at least 2 doses administered, or
	• Fewer than 2 doses or the age-appropriate number of doses or an unknown number of doses for each of the pneumococcal and pentavalent conjugate vaccines
**ITIP2 and ITIP3 eligibility criteria**	
Inclusion criteria	• 2-59 months of age
	• Cough <14 days or difficulty breathing
	• Chest indrawing
	• ITIP3 chest-indrawing pneumonia cohort: excluded from enrollment in ITIP2 clinical trial due to the presence of any of the following:
	- Severe respiratory distress
	- Hypoxemia
	- Hemoglobin <8.0 g/dL, if a positive mRDT
	- Severe acute malnutrition
	- Severe malaria (ie, positive mRDT with any WHO IMCI general danger sign, stiff neck, abnormal bleeding, clinical jaundice, or hemoglobinuria)
	- HIV seropositivity or HIV exposure
	• Ability and willingness of child’s caregiver to provide informed consent and to be available for follow-up for the planned duration of the study, including accepting a home visit if he/she fails to return for a scheduled study follow-up visit
Exclusion criteria	• Stridor when calm
	• Possible tuberculosis (coughing for more than 14 days)
	• Hemoglobin <8.0 g/dL, if a negative mRDT
	• Known allergy to penicillin or amoxicillin
	• Receipt of an antibiotic treatment in the 48 hours prior to the study
	• Living outside the study area
	• Any medical or psychosocial condition or circumstance that, in the opinion of the investigators, would interfere with the conduct of the study or for which study participation might jeopardize the child’s health
	• Participation in a clinical study of an investigational product within 12 weeks prior to enrollment or planning to begin participation during this study
	• Prior participation in ITIP study during a previous pneumonia diagnosis
	• ITIP2 clinical trial only:
	- Severe respiratory distress
	- Hypoxemia
	- Hemoglobin <8.0 g/dL, if a positive mRDT
	- Severe acute malnutrition
	- Severe malaria (ie, positive mRDT with any WHO IMCI general danger sign, stiff neck, abnormal bleeding, clinical jaundice, or hemoglobinuria)
	▪ HIV seropositivity or HIV exposure
**ITIP2 and ITIP3 clinical outcomes**	
Treatment failure*	For both ITIP2 and ITIP3, development of very fast breathing, chest indrawing, severe respiratory distress, hypoxemia, WHO IMCI general danger signs, fever, or change in antibiotics:
	• For ITIP2, prior to or on Day 6
	• For ITIP3, on Day 6 only
	For ITIP2 only, missing 3 or more study drug doses due to vomiting
	For both ITIP2 and ITIP3, death prior to or on Day 6
Clinically cured†	For both ITIP2 and ITIP3, absence of fast-breathing pneumonia, very fast breathing for age, chest indrawing, severe respiratory distress, hypoxemia, WHO IMCI general danger signs, and fever at Day 14:
	• Cured but failed initial antibiotic treatment regimen
	• Cured and did not fail initial antibiotic treatment regimen
Not clinically cured†	Day 14:
	• Deteriorating
	• Stable (not improving or deteriorating, prognosis unclear)
	On or prior to Day 16:
	• Death

ITIP2 – Innovative Treatments in Pneumonia chest-indrawing pneumonia clinical trial, ITIP3 – Innovative Treatments in Pneumonia prospective observational study

*Final visits for treatment failure assessment (Day 6) may have occurred 1 day later (Day 7).

†Clinically cured status is assessed from the last visit that may have occurred 2 days earlier to 2 days later than the scheduled day (ie, on actual Days 12 through 16).

The studies were conducted in accordance with the International Conference on Harmonisation, Good Clinical Practice and the Declaration of Helsinki 2008, and were approved by the Western Institutional Review Board (Washington, USA); the College of Medicine Research and Ethics Committee (Blantyre, Malawi); and the Malawi Pharmacy, Medicines and Poisons Board; and were registered with ClinicalTrials.gov (NCT02678195 and NCT02960919).

### Study procedures

On Day 1, after enrollment informed consent was obtained from the caregiver by study staff, ITIP eligible children received a physical examination, and information regarding their medical and vaccination history along with additional socio-demographic information was collected. If qualifying for admission, children were admitted to KCH where follow-up visits also occurred once discharged.

ITIP2 children were randomized to either receive 3- or 5-day oral amoxicillin. ITIP3 children received standard-of-care for their illnesses per Malawian guidelines and KCH protocols which typically included 5-day oral amoxicillin or, for severe pneumonia, parenteral ampicillin/penicillin and gentamicin. Children were followed by study staff with scheduled in-person visits on Days 2 (while hospitalized), 4 (ITIP2 only), 6, and 14, and by phone on Day 30 (ITIP3 only). In case of a no-show at scheduled follow-up visits, a home visit was conducted. In ITIP3, if a phone call on Day 30 was not possible, study staff conducted a home visit to obtain Day 30 outcome information. Nether study included laboratory or radiologic examinations.

### Study outcomes

The primary endpoints in ITIP3 included clinical outcomes of children treated for pneumonia by Day 6 and clinical cure rates at Day 14 (which were defined independently of the availability of Day 6 outcome). Select ITIP3 subgroups categorized by individual comorbidity (HIV infection or exposure, malaria, MAM or SAM) were compared to children in the standard-of-care arm of the ITIP2 CIP trial. Secondary endpoints included treatment responses (vital signs, oxygen saturation, length of hospital stay) and proportion of children who were re-hospitalized or died. In this analysis we compared treatment failure (TF) and clinical outcomes in ITIP3′s CIP cohort to TF and clinical outcomes in ITIP2’s CIP trial population. ITIP3 children were followed for 30 days post-enrollment. ITIP2’s population was followed for 14 days post-enrollment. Because ITIP2 was a clinical trial with randomized treatments whereas ITIP3 was an observational study with standard-of-care treatments, study visits were more frequent for ITIP2 than ITIP3 during the first 14 days. Development of very fast breathing is included here in the TF definition for ITIP2 and ITIP3, but was not included in the TF definition for ITIP2 protocol and primary manuscript [4].

### Statistical analysis

χ^2^ statistics, Fisher exact tests, and *t* tests were used to compare baseline characteristics of ITIP2 and ITIP3 CIP children. Generalized linear models with robust standard errors were used to compare the percentages (absolute risk differences) of TF by Day 6 and of clinical cure status at Day 14 among ITIP3 CIP children with at least 1 comorbidity at enrollment to ITIP2 children. The ITIP2 3- and 5-day amoxicillin treatment arms were separately compared to subgroups of ITIP3 children with HIV infection or exposure, laboratory result positive or severe malaria, MAM or SAM, or anemia, respectively. For the comparisons of ITIP3 children with severe malaria or MAM or SAM, ITIP2 children who had non-severe malaria or MAM were excluded to allow comparisons to be made between ITIP3 CIP children with a specific comorbidity, and ITIP2 children without that comorbidity. Comparisons were also made based on whether or not WHO general or respiratory danger signs (ie, lethargy or unconsciousness, convulsions, vomiting everything, inability to drink or feed, grunting, nasal flaring, and/or head nodding) were present beyond chest indrawing among ITIP3 children with comorbidities. We adjusted for sex, age category (2-6, 7-11, 12-23, or 24-59 months), weight, and vaccination status in each model. Differences in clinical outcome rates by vaccination status were estimated using similar models, adjusting for the presence or absence of each of the comorbidities and the presence or absence of danger signs at enrollment. Tests were performed as two-sided with alpha = 0.05. No adjustments were made for multiple comparisons because of the study’s observational and exploratory nature.

### Role of the funding source

The funder provided some input on study design.

## RESULTS

**Figure 1:**
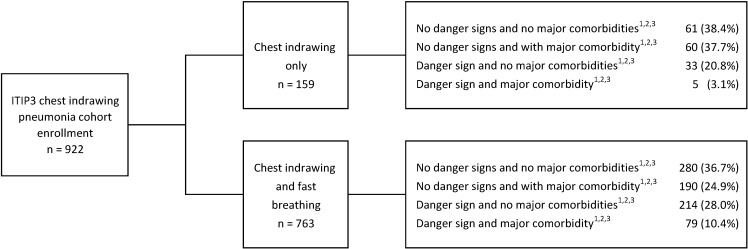
ITIP3 chest-indrawing pneumonia cohort enrollment by comorbidities. *Subgroups are based on data from screening/enrollment form. †Danger signs defined as one of more of the following: lethargy or unconsciousness, convulsions, vomiting everything, inability to drink or feed, grunting, nasal flaring, and/or head nodding. ‡Major comorbidity defined as 1 or more of the following: HIV: positive HIV-1 rapid diagnostic test or HIV exposure; Malaria: positive malaria rapid diagnostic test with or without any danger signs, stiff neck, abnormal bleeding, clinical jaundice, or hemoglobinuria; Malnutrition: mid-upper arm circumference <12.5 cm or weight-for-height z-score<-2; Anemia: hemoglobin <8 g/dL.

From October 19, 2016 to June 13, 2018, 922 ITIP3 children with CIP were enrolled, 763 with and 159 without fast breathing (**Figure 1**). Of the 922 children in the ITIP3 CIP cohort, 298 had at least 1 comorbidity and 331 (247 + 84) had at least 1 danger sign, including 70 children with both (data not shown). In ITIP3, TF by Day 6 and clinical outcome at Day 14 assessment data were available for 892 (96.7%) and 880 (95.4%) CIP children, respectively. In ITIP2, 3000 CIP children were enrolled; 1497 randomized to receive 3-day amoxicillin and 1503 randomized to receive 5-day amoxicillin. One child with HIV infection or exposure inappropriately enrolled in ITIP2 and randomized to the 3-day arm was omitted from these analyses, resulting in data being used from 2999 children. TF by Day 6 assessment data in ITIP2 were available for 1441 (96.3%) and 1456 (96.9%) children in the 3- and 5-day arms, respectively; clinically cured at Day 14 outcome data in ITIP2 were available for 1420 (94.9%) and 1440 (95.8%) children in the 3- and 5-day arms, respectively.

When comparing the ITIP3 CIP cohort to ITIP2, there were few differences in baseline characteristics (**Table 2**). The ITIP3 CIP cohort had slightly more male children (58.9%) than ITIP2 (55.1%; *P* = 0.048). ITIP3 children were generally younger than those in ITIP2 (44.0% of children aged 2-6 months in ITIP3 vs 38.5% in ITIP2; *P* = 0.01). Respiratory rates differed between the ITIP3 CIP cohort and ITIP2, with more children in ITIP3 than in ITIP2 meeting the very fast breathing criteria (among children aged 2-11 months, 13.5% in ITIP3 vs 2.5% in ITIP2; among children aged 12-59 months, 29.0% in ITIP3 vs 7.2% in ITIP2; *P* < 0.01). Oxygen saturation levels also differed between the ITIP3 CIP cohort and ITIP2, with hypoxemia more common in ITIP3 than in ITIP2 (0.3% vs 0%). Hypoxemia was an exclusion criterion for ITIP2. Fevers appeared to be similarly common between the ITIP3 CIP cohort and ITIP2 (29.8% vs 30.9%; *P* = 0.57). The average heart rate was slightly higher in the ITIP3 CIP cohort than ITIP2 (156.8 vs 150.3 beats per minute; *P* < 0.01).

**Table 2:** ITIP3 chest-indrawing cohort and ITIP2 baseline characteristics by (not mutually exclusive) comorbidity groups

	ITIP3 chest-indrawing pneumonia cohort					ITIP2 overall	*P*-value comparing ITIP3 overall to ITIP2 overall
	HIV*,†	Malaria*,‡	Malnutrition*,§	Anemia*,‖	**Overall**
	**(n = 139)**	**(n = 143)**	**(n = 83)**	**(n = 76)**	**(n = 922)**	**(n = 2999)**
Age (months):							
Mean (SD)	9.1 (7.6)	16.5 (13.3)	5.9 (5.3)	14.2 (11.2)	12.3 (11.9)	13.6 (12.3)	0.01††
2-6, n (%)	65 (46.8%)	38 (26.6%)	61 (73.5%)	20 (26.3%)	406 (44.0%)	1155 (38.5%)	
7-11, n (%)	41 (29.5%)	29 (20.3%)	10 (12.0%)	22 (28.9%)	178 (19.3%)	581 (19.4%)	
12-23, n (%)	29 (20.9%)	44 (30.8%)	11 (13.3%)	23 (30.3%)	200 (21.7%)	712 (23.7%)	
24-59, n (%)	4 (2.9%)	32 (22.4%)	1 (1.2%)	11 (14.5%)	138 (15.0%)	551 (18.4%)	
Sex:							
Male, n (%)	79 (56.8%)	69 (48.3%)	40 (48.2%)	38 (50.0%)	543 (58.9%)	1653 (55.1%)	0.048††
Female, n (%)	60 (43.2%)	74 (51.7%)	43 (51.8%)	38 (50.0%)	379 (41.1%)	1346 (44.9%)	
Respiratory rate¶ (breaths/min):							
2-11 months old:							
<50, n (%)	24 (22.6%)	6 (9.0%)	6 (8.5%)	2 (4.8%)	78 (13.4%)	603 (34.7%)	<0.01††
50-59, n (%)	45 (42.5%)	25 (37.3%)	31 (43.7%)	22 (52.4%)	254 (43.5%)	754 (43.4%)	
60-69, n (%)	29 (27.4%)	22 (32.8%)	25 (35.2%)	10 (23.8%)	173 (29.6%)	335 (19.3%)	
≥70, n (%)	8 (7.5%)	14 (20.9%)	9 (12.7%)	8 (19.0%)	79 (13.5%)	44 (2.5%)	
12-59 months old:							
<40, n (%)	5 (15.2%)	4 (5.3%)	0 (0.0%)	1 (2.9%)	25 (7.4%)	324 (25.7%)	<0.01††
40-49, n (%)	9 (27.3%)	16 (21.1%)	1 (8.3%)	9 (26.5%)	91 (26.9%)	513 (40.6%)	
50-59, n (%)	13 (39.4%)	32 (42.1%)	8 (66.7%)	11 (32.4%)	124 (36.7%)	335 (26.5%)	
≥60, n (%)	6 (18.2%)	24 (31.6%)	3 (25.0%)	13 (38.2%)	98 (29.0%)	91 (7.2%)	
Oxygen saturation** (%):							
<90, n (%)	0 (0.0%)	0 (0.0%)	0 (0.0%)	0 (0.0%)	3 (0.3%)	0 (0.0%)	<0.01††
90-92, n (%)	1 (0.7%)	0 (0.0%)	0 (0.0%)	0 (0.0%)	10 (1.1%)	12 (0.4%)	
≥93, n (%)	138 (99.3%)	143 (100.0%)	83 (100.0%)	76 (100.0%)	909 (98.6%)	2987 (99.6%)	
Axillary temperature¶ (°C):							
≥38, n (%)	44 (31.7%)	74 (51.7%)	15 (18.1%)	46 (60.5%)	275 (29.8%)	926 (30.9%)	0.57††
<38, n (%)	95 (68.3%)	69 (48.3%)	68 (81.9%)	30 (39.5%)	647 (70.2%)	2073 (69.1%)	
Heart rate** (beats/min):							
Mean (SD)	153.8 (13.9)	163.3 (17.9)	159.3 (16.0)	166.1 (16.2)	156.8 (15.6)	150.3 (16.2)	<0.01‡‡
Minimum, Maximum	124, 207	118, 216	124, 207	138, 202	90, 216	92, 212	

ITIP2 – Innovative Treatments in Pneumonia chest-indrawing pneumonia clinical trial, ITIP3 – Innovative Treatments in Pneumonia prospective observational study, n – number, SD – standard deviation

*Subgroups are based on data from screening/enrollment form and are non-exclusive.

†HIV defined as a positive HIV-1 rapid diagnostic test or HIV exposure.

‡Malaria defined as a positive malaria rapid diagnostic test with or without any danger signs, stiff neck, abnormal bleeding, clinical jaundice, or hemoglobinuria.

§Malnutrition defined as mid-upper arm circumference <12.5 cm or weight-for-height z-score<-2.

**‖**Anemia defined as a hemoglobin <8 g/dL.

¶Maximum value between screening and enrollment.

**Minimum value between screening and enrollment.

††Based on the comparison of counts using the χ^2^ or Fisher exact test.

‡‡Based on the comparison of means using the *t* test.

Among the ITIP3 CIP cohort subgroups categorized by individual comorbidity, observed TF rates by Day 6 were higher in those with danger signs (ranging from 17.3% for those with malaria to 31.2% for those with HIV infection or exposure) than in those without danger signs (ranging from 6.6% for those with HIV infection or exposure to 11.7% for those with MAM or SAM) (**Table 3**). When comparing ITIP3 children categorized by individual comorbidity in the CIP cohort to ITIP2 children without each respective comorbidity, adjusted differences were generally higher among ITIP3 children with a comorbidity than among ITIP2 children without the respective comorbidity. The differences were only statistically significantly higher for ITIP3 children with HIV infection or exposure and danger signs ( ~ 25% higher) and for ITIP3 children with malaria and danger signs ( ~ 12% higher). Children in ITIP3 with HIV infection or exposure and without danger signs had slightly lower TF rates compared to ITIP2 children in the 3-day arm (-0.6%). When subdividing malaria and acute malnutrition in ITIP3 by severity, rather than by the presence or absence of danger signs, TF rates were 5.0%-7.4% higher among those with malaria or MAM and 8.4%-9.4% higher among those with severe malaria or SAM than among those without these comorbidities, but none of these adjusted differences were statistically significant (data not shown).

**Table 3:** Treatment failure rates* with adjusted† differences and 95% confidence intervals

ITIP3 chest-indrawing pneumonia cohort treatment failure rates		ITIP2 treatment failure rates in reference groups		ITIP3 vs ITIP2 adjusted differences in treatment failure rates	
**3-day amoxicillin**	**5-day amoxicillin**	**3-day amoxicillin**	**5-day amoxicillin**
HIV§,‡					
With danger sign	5/16 (31.2%)	88/1441 (6.1%)^††^	77/1456 (5.3%)^††^	24.8 (2.3 to 47.4)	25.6 (3.1 to 48.2)
With no danger signs	8/121 (6.6%)			-0.6 (-5.3 to 4.1)	0.4 (-4.3 to 5.0)
Malaria**‖**:					
With danger sign	9/52 (17.3%)	83/1314 (6.3%)^††^	72/1320 (5.5%)^††^	11.5 (1.2 to 21.9)	12.0 (1.6 to 22.4)
With no danger signs	7/83 (8.4%)			1.7 (-4.5 to 8.0)	2.4 (-3.8 to 8.6)
Malnutrition¶,‡					
With danger sign	5/20 (25.0%)	81/1396 (5.8%)^††^	73/1409 (5.2%)^††^	17.8 (-1.1 to 36.6)	17.2 (-1.5 to 35.9)
With no danger signs	7/60 (11.7%)			4.6 (-3.7 to 13.0)	4.7 (-3.7 to 13.0)
Anemia**,‡					
With danger sign	3/16 (18.8%)	88/1441 (6.1%)^††^	77/1456 (5.3%)^††^	12.2 (-6.6 to 31.0)	12.7 (-6.1 to 31.5)
With no danger signs	5/55 (9.1%)			2.1 (-5.8 to 10.0)	2.8 (-5.0 to 10.7)

ITIP2 – Innovative Treatments in Pneumonia chest-indrawing pneumonia clinical trial, ITIP3 - Innovative Treatments in Pneumonia prospective observational study

*Treatment failure rates are a percentage of those with a known treatment failure status.

†Adjusted for sex, age-category, weight, vaccination status.

‡Subgroup based on data from screening/enrollment form.

§HIV defined as a positive HIV-1 rapid diagnostic test or HIV exposure.

**‖**Malaria defined as a positive malaria rapid diagnostic test with or without any danger signs, stiff neck, abnormal bleeding, clinical jaundice, or hemoglobinuria.

¶Malnutrition defined as mid-upper arm circumference <12.5 cm or weight-for-height z-score<-2.

**Anemia defined as a hemoglobin <8 g/dL.

††ITIP2 reference group consists of children without each major comorbidity of interest for ITIP3 children.

Among the ITIP3 CIP cohort subgroups categorized by individual comorbidity, observed rates of those not clinically cured at Day 14 ranged from 0%-12.5% for children who also had danger signs, and ranged from 7.3%-20.7% for children who did not have danger signs (**Table 4**). Children in ITIP3 with HIV infection or exposure or malaria had about 6%-10% higher adjusted rates of not being clinically cured compared to ITIP2 children without each respective comorbidity, with rates higher among those with danger signs compared to those without danger signs (with some of the adjusted differences statistically significant). By contrast, children with MAM or SAM in ITIP3 had significantly higher adjusted rates of those not clinically cured if they had no danger signs (though the number of children with MAM or SAM and danger signs was relatively small). Of note, all of the 16 children with anemia and danger signs in ITIP3 were clinically cured which resulted in adjusted negative differences when compared to ITIP2 children. When subdividing malaria and acute malnutrition in ITIP3 by severity, rather than by the presence or absence of danger signs, estimated adjusted differences were higher (ranging from 6.5%-20.0%; some not statistically significant) in ITIP3 than in ITIP2, with about 20% (statistically significantly) higher rates of those not clinically cured for children with SAM in ITIP3 vs those in ITIP2 (data not shown).

**Table 4:** Rates of those not clinically cured* with adjusted† differences and 95% confidence intervals

ITIP3 vs ITIP2 adjusted differences in clinical cure rates		ITIP2 rates of those not clinically cured in reference groups		ITIP3 vs ITIP2 adjusted differences in clinical cure rates	
		**3-day amoxicillin**	**5-day amoxicillin**	**3-day amoxicillin**	**5-day amoxicillin**
HIV§,‡					
With danger sign	2/16 (12.5%)	58/1420 (4.1%)††	39/1440 (2.7%)††	8.4 (-6.6 to 23.5)	9.7 (-5.7 to 25.1)
With no danger signs	15/121 (12.4%)			7.7 (1.9 to 13.6)	9.4 (3.6 to 15.2)
Malaria**‖**,‡					
With danger sign	6/50 (12.0%)	50/1293 (3.9%)††	36/1304 (2.8%)††	7.9 (-1.2 to 17.0)	8.7 (-0.4 to 17.8)
With no danger signs	9/83 (10.8%)			6.5 (-0.2 to 13.2)	7.7 (1.0 to 14.4)
Malnutrition¶,‡					
With danger sign	2/19 (10.5%)	51/1377 (3.7%)††	35/1395 (2.5%)††	3.9 (-9.9 to 17.8)	6.1 (-7.5 to 19.8)
With no danger signs	12/58 (20.7%)			15.3 (4.9 to 25.8)	17.0 (6.6 to 27.3)
Anemia:**,‡					
With danger sign	0/16 (0.0%)	58/1420 (4.1%)††	39/1440 (2.7%)††	-4.3 (-6.3 to -2.3)	-3.1 (-4.9 to -1.4)
With no danger signs	4/55 (7.3%)			2.5 (-4.4 to 9.4)	4.1 (-2.8 to 11.0)

ITIP2 – Innovative Treatments in Pneumonia chest-indrawing pneumonia clinical trial, ITIP3 - Innovative Treatments in Pneumonia prospective observational study

*Rates of those not clinically cured at Day 14 are a percentage of those with a known clinical cure status.

†Adjusted for sex, age-category, weight, vaccination status.

‡Subgroup based on data from screening/enrollment form.

§HIV defined as a positive HIV-1 rapid diagnostic test or HIV exposure.

**‖**Malaria defined as a positive malaria rapid diagnostic test with or without any danger signs, stiff neck, abnormal bleeding, clinical jaundice, or hemoglobinuria.

¶Malnutrition defined as mid-upper arm circumference <12.5 cm or weight-for-height z-score<-2.

**Anemia defined as a hemoglobin <8 g/dL.

††ITIP2 reference group consists of children without each major comorbidity of interest for ITIP3 children.

**Figure 2:**
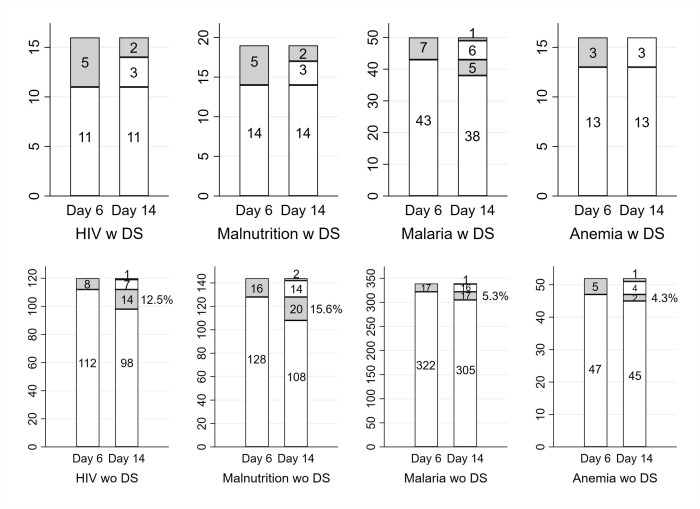
Treatment failure status (yes = gray/ no = white) by Day 6 and clinically cured status (no = gray/ yes = white) by Day 14 for pre-selected comorbidities (HIV infection or exposure; moderate or severe acute malnutrition; malaria; and anemia) stratified by whether (w) or not (wo) children presented with danger signs (DS)

We also considered the rate of those not clinically cured at Day 14 by TF status by Day 6 for those with danger signs and those without. We focused for this comparison on ITIP3 children with HIV infection or exposure and on ITIP3 children with MAM or SAM, and also included ITIP2 children with MAM. Among those ITIP3 children with danger signs at enrollment, in addition to having either HIV infection or exposure or MAM or SAM, all were clinically cured at Day 14 if they did not have TF by Day 6. Of note, ITIP3 children with danger signs at enrollment typically received antibiotics for at least 5 days and potentially additional pneumonia treatment after Day 6. By contrast, among those children without danger signs at enrollment but with HIV infection or exposure or MAM or SAM, 12.5% and 15.6% were not clinically cured at Day 14 if they were without TF by Day 6 (**Figure 2**). ITIP2 and ITIP3 children with comorbidities but no danger signs at enrollment initially received antibiotics, but then typically no further pneumonia treatment after Day 6 if they did not have TF by Day 6.

Comparing children with at least two age-appropriate pentavalent (including *Haemophilus influenzae* type b) vaccine doses to those with fewer or unknown doses showed mixed estimated adjusted differences in TF and clinical cure rates. Having at least 2 age-appropriate pentavalent vaccinations was associated with a 6.0% increased rate of those not clinically cured compared to having fewer or unknown numbers of vaccinations in the combined cohort of children in ITIP3 and in the ITIP2 3-day arm. Conversely, having at least 2 age-appropriate pneumococcal vaccinations was associated with a -6.2% decreased rate of those not clinically cured compared to having fewer or unknown numbers of vaccinations in the combined cohort of children in ITIP3 and in the ITIP2 3-day arm; none of these differences were statistically significant (**Table 5**). Other estimated differences for pentavalent and pneumococcal vaccinations were smaller in magnitude and also not statistically significant. Note that vaccination records were at least partially unknown for many children, particularly those at least 14 weeks old, for whom approximately 26% had entirely unknown numbers of pentavalent and pneumococcal conjugate vaccine doses (Appendix S1 in the **Online Supplementary Document**).

**Table 5:** Adjusted* differences with 95% confidence intervals in clinical outcome rates by pentavalent and pneumococcal conjugate vaccination status† in combined cohorts

	Adjusted difference in treatment failure rates (95% CI) based on children in ITIP3 and ITIP2 3-day amoxicillin arm	Adjusted difference in treatment failure rates (95% CI) based on children in ITIP3 and ITIP2 5-day amoxicillin arm	Adjusted difference in rates of those not clinically cured (95% CI) based on children in ITIP3 and ITIP2 3-day amoxicillin arm	Adjusted difference in rates of those not clinically cured (95% CI) based on children in ITIP3 and ITIP2 5-day amoxicillin arm
Pentavalent (includes *Haemophilus influenzae* type b) vaccinations	-1.6 (-10.4 to 7.3)	1.8 (-7.9 to 11.5)	6.0 (-3.3 to 15.4)	0.6 (-4.9 to 6.2)
Pneumococcal conjugate vaccinations	-1.0 (-9.8 to 7.8)	-4.6 (-14.3 to 5)	-6.2 (-15.6 to 3.2)	-0.5 (-6.0 to 5.0)

ITIP2 – Innovative Treatments in Pneumonia chest-indrawing pneumonia clinical trial, ITIP3 – Innovative Treatments in Pneumonia prospective observational study, CI – confidence interval

*Adjusted for sex, age-category, weight, presence or absence of danger signs, and presence or absence of comorbidities at enrollment.

†Children with at least 2 age-appropriate vaccination doses are compared to children with fewer or unknown numbers of vaccination doses.

No deaths occurred among ITIP3 or ITIP2 children prior to TF assessment. Three deaths in ITIP2 and 3 deaths in ITIP3 were recorded between Days 6 to 16, and no later deaths were recorded in either arm.

## DISCUSSION

Children in ITIP3′s CIP cohort with comorbidities but without danger signs did not have statistically significant higher TF rates than those in the ITIP2 CIP trial. However, ITIP3 CIP children with HIV infection or exposure, malaria, or MAM or SAM had higher rates of those not clinically cured at Day 14 when compared to children without these comorbidities. If CIP children also demonstrated danger signs in combination with a comorbidity, these children had considerably higher TF rates, ranging between 11.5% and 25.6%. With the exception of anemia, rates of those not clinically cured were higher for those with comorbidities among ITIP3 children compared to those without comorbidities in ITIP2, regardless of whether or not they had danger signs at enrollment. It may be surprising that these rates were either comparable or higher among children without danger signs than among those with danger signs. However, children with danger signs initially receive more intensive treatment than children without danger signs. Children without danger signs who have no TF by Day 6 receive no further pneumonia treatment post Day 6.

Over a third (36.2%) of ITIP3 CIP children had ≥1 comorbidity, with HIV infection or exposure and malaria the most common comorbidities. Nasal flaring (31.1%), head nodding (10.1%), and grunting (7.8%) were the most common danger signs, although the percentage of CIP children with both comorbidity and any danger sign was low (9.1%). Among children with danger signs, for all individual comorbidities, the percentage of children with TF (ranging from 17.3% for malaria to 31.2% for HIV infection or exposure) was higher than the percentage of children who were not clinically cured (ranging from 0.0% for anemia to 12.5% for HIV infection or exposure). The opposite is true, with the exception of anemia, for children with comorbidities but without danger signs; the percentage of children with TF is smaller than the percentage of children who were not clinically cured (6.6% vs 12.4% for HIV infection or exposure; 8.4% vs 10.8% for malaria; 11.7% vs 20.7% for malnutrition; 9.1% vs 7.3% for anemia). More specifically, when comparing clinical cure status by absence or presence of TF within each subgroup with a comorbidity, there was a noticeable percentage of children with comorbidities who neither had danger signs at enrollment nor TF by Day 6, who were not clinically cured at Day 14. Children with TF by Day 6 and those with danger signs at enrollment received additional treatment. It appears that this additional treatment worked for most. Nearly all children with danger signs at enrollment who appeared well and did not have TF by Day 6 were still doing well at Day 14, with the only exception being a subset of children with malaria. However, there were children with comorbidities who did not exhibit any danger signs at enrollment who appeared well by Day 6 and thus did not receive further pneumonia treatment beyond Day 6, who were not well at Day 14.

These results suggest that some children with comorbidities and no danger signs are at higher risk for not being clinically cured at Day 14. With an absence of TF by Day 6, in general practice children typically will not be seen by a health care provider by Day 6. However, it may be advisable that children with comorbidities such as HIV infection or exposure or malnutrition potentially should be seen by a health care provider by Day 6 even in the absence of TF. Other diagnostic approaches such as lung ultrasound could potentially identify children who would benefit from continued pneumonia treatment to ensure clinical cure at Day 14.

Children in ITIP3’s CIP cohort who were excluded from the ITIP2 CIP trial due to comorbidities and/or danger signs demonstrated lower clinical cure rates. It may be that these higher risk children were not able to seek care before their pneumonia progressed to CIP.

Adjusted risk differences for pneumococcal conjugate vaccinations were consistent with somewhat increased protection but the adjusted risk differences for pentavalent vaccinations were mixed and inconsistent. None of the adjusted risk differences for pneumococcal conjugate or pentavalent vaccinations were statistically significant, and a substantial amount of missing data may have biased or diluted any potential effect of vaccinations.

### Strengths and limitations

A limitation to this comparison of ITIP3’s CIP cohort to the ITIP2 trial included the small number of children representing any ITIP3 subgroup categorized by individual comorbidity, and then within those ITIP3 subgroups categorized by individual comorbidity, the small number who exhibited danger signs. We also focused on selected common, major comorbidities, and not all potential comorbidities, such as a history of being born preterm or low birthweight. Other limitations were different treatment regimens used and the lack of structured follow-up in ITIP3 compared to ITIP2 (study visits were more frequent for ITIP2 than for ITIP3 during the first 14 days), which limited the information available for ITIP3 regarding clinical course and made direct comparisons difficult. These structural differences were intentional because ITIP2 was a clinical trial with randomized treatments and close follow-up, and ITIP3 was an observational study using standard-of-care treatments with some additional follow-up. We applied the relevant WHO management guidelines and TF criteria specific to each of the ITIP2 and ITIP3 groups. We were unable to apply the same TF criteria to both groups given that the guidelines themselves are different. We chose to apply WHO guidelines for diagnosing and treating pneumonia to these groups because it was important for our results to be representative of real-world care, and these are the guidelines that health care providers use during routine care conditions. Finally, this is a complete case analysis and other more extensive sensitivity analyses were not performed due to the exploratory and observational nature of the analysis.

## CONCLUSION

ITIP3 CIP children who were excluded from the concurrent ITIP2 trial due to comorbidities and danger signs demonstrated similar TF rates, but higher rates of those not clinically cured at Day 14. In particular, for each of the comorbidity subgroups, some children with comorbidities but without danger signs appeared well by Day 6, but not at Day 14. Given that, it may be important to consider more intensive follow-up of CIP children with comorbidities who do not have danger signs.

## Additional material

Online Supplementary Document
