# Supplementary material for: Malawian children with chest-indrawing pneumonia with and without comorbidities or danger signs
Source: J Glob Health. 2021 Mar 7;11:04016. doi: 10.7189/jogh.11.04016 (PMC7979154; doi:10.7189/jogh.11.04016)
Supplement: Online Supplementary Document [file jogh-11-04016-s001.pdf]

Appendix S1. ITIP3 chest-indrawing pneumonia cohort and ITIP2 pentavalent and pneumococcal vaccinations

	ITIP3 chest-indrawing pneumonia cohort n (%)			ITIP2 3-day amoxicillin n (%)			ITIP2 5-day amoxicillin n (%)		
	< 10 weeks	10 - 14 weeks	≥ 14 weeks	< 10 weeks	10 - 14 weeks	≥ 14 weeks	< 10 weeks	10 - 14 weeks	≥ 14 weeks
	n=34	n=109	n=779	n=45	n=126	n=1325	n=36	n=120	n=1347
Pentavalent vaccine (includes *Haemophilus influenzae* type b conjugate vaccine)									
Received age-appropriate number of doses,* n (%)	28 (82.4%)	48 (44..0%)	469 (60.2%)	40 (88.9%)	66 (52.4%)	842 (63.5%)	32 (88.9%)	66 (55.0%)	854 (63.4%)
All doses unknown, n (%)	1 (2.9%)	3 (2.8%)	212 (27.2%)	3 (6.7%)	2 (1.6%)	351 (26.5%)	2 (5.6%)	0 (0.0%)	359 (26.7%)
Some doses missed or unknown, n (%)	5 (14.7%)	58 (53.2%)	98 (12.6%)	2 (4.4%)	58 (46.0%)	132 (10.0%)	2 (5.6%)	54 (45.0%)	134 (9.9%)
Received 2 doses, n (%)	0 (0.0%)	0 (0.0%)	72 (9.2%)	0 (0.0%)	0 (0.0%)	106 (8.0%)	0 (0.0%)	0 (0.0%)	108 (8.0%)
Received 1 dose, n (%)	0 (0.0%)	49 (45.0%)	23 (3.0%)	0 (0.0%)	56 (44.4%)	21 (1.6%)	0 (0.0%)	50 (41.7%)	22 (1.6%)
Received 0 doses, n (%)	5 (14.7%)	9 (8.3%)	3 (0.4%)	2 (4.4%)	2 (1.6%)	5 (0.4%)	2 (5.6%)	4 (3.3%)	4 (0.3%)
Pneumococcal conjugate vaccine									
Received age-appropriate number of doses,* n (%)	27 (79.4%)	48 (44.0%)	463 (59.4%)	40 (88.9%)	66 (52.4%)	835 (63.0%)	32 (88.9%)	67 (55.8%)	853 (63.3%)
All doses unknown, n (%)	1 (2.9%)	3 (2.8%)	212 (27.2%)	3 (6.7%)	2 (1.6%)	352 (26.6%)	2 (5.6%)	0 (0.0%)	360 (26.7%)
Some doses missed or unknown, n (%)	6 (17.6%)	58 (53.2%)	104 (13.4%)	2 (4.4%)	58 (46.0%)	138 (10.4%)	2 (5.6%)	53 (44.2%)	134 (9.9%)
Received 2 doses, n (%)	0 (0.0%)	0 (0.0%)	77 (9.9%)	0 (0.0%)	0 (0.0%)	109 (8.2%)	0 (0.0%)	0 (0.0%)	110 (8.2%)
Received 1 dose, n (%)	0 (0.0%)	49 (45.0%)	24 (3.1%)	0 (0.0%)	56 (44.4%)	23 (1.7%)	0 (0.0%)	49 (40.8%)	21 (1.6%)
Received 0 doses, n (%)	6 (17.6%)	9 (8.3%)	3 (0.4%)	2 (4.4%)	2 (1.6%)	6 (0.5%)	2 (5.6%)	4 (3.3%)	3 (0.2%)

* One dose if at least 6 weeks and up to 10 weeks old, 2 doses if at least 10 weeks and up to 14 weeks old, 3 doses if at least 14 weeks old
